# Supplementary material for: Interaction paths promote module integration and network-level robustness of spliceosome to cascading effects
Source: Sci Rep. 2018 Nov 28;8:17441. doi: 10.1038/s41598-018-35160-6 (PMC6261937; doi:10.1038/s41598-018-35160-6)
Supplement: Supplementary file 1 — Supplementary information [file 41598_2018_35160_MOESM1_ESM.docx]

Supporting Information

**Interaction paths promote module integration and network-level robustness of spliceosome to cascading effects**

**Paulo R. Guimarães Jr**^1^**, Mathias M. Pires**^2^**, Maurício Cantor**^3,4^**, Patricia P. Coltri**^5^

^1^ Departamento de Ecologia, Instituto de Biociências, Universidade de São Paulo, Rua do Matão, Travessa 14, 05508-900, São Paulo, SP, Brazil;

^2^ Departamento de Biologia Animal, Instituto de Biologia, Universidade Estadual de Campinas, Rua Monteiro Lobato 255, 13083-862, Campinas, SP, Brazil;

^3^ Departamento de Ecologia e Zoologia, Centro de Ciências Biológicas, Universidade Federal de Santa Catarina, Trindade, Caixa Postal 5102, CEP 88040- 970, Florianópolis, SC, Brazil;

^4^ Centro de Estudos do Mar, Universidade Federal do Paraná, Av. Beira-mar, s/n, Caixa Postal 61, CEP 83255-976, Pontal do Paraná, PR, Brazil;

^5^ Departamento de Biologia Celular e do Desenvolvimento, Instituto de Ciências Biomédicas, Universidade de São Paulo, Av Prof Lineu Prestes, 1524, ICB-I, 05508-000, São Paulo, SP, Brazil.

**Table of Contents**

1. Table S1 01
2. Sensitivity analyses: spliceosome network with low cutoff (0.15) 02
3. Sensitivity analyses: alternative null models 03

**Table S1.** The structure of the spliceosome network assuming low (0.15) and high (0.5) cutoff values for the reliability values. Source for all descriptor values but clustering coefficient and average shortest path length: Pires et al. (2015; doi: 10.1038/srep14865). Clustering coefficient and average shortest path length were computed using a MATLAB script available upon request. Definitions of all descriptors are available at the main text.

| **Network descriptor** | **Low cutoff** | **High cutoff** |
| --- | --- | --- |
| Number of interactions | 2538 | 881 |
| Connectance | 0.483 | 0.168 |
| Average degree | 49.28 ± 24.08 | 17.11 ± 13.04 |
| Modularity (*Q*) | 0.12 | 0.32 |
| Number of modules | 3 | 4 |
| Nestedness (NODF) | 0.75 | 0.38 |
| Clustering coefficient | 0.769 | 0.566 |
| Average shortest path length | 1.54 | 2.03 |

1. **Sensitivity analyses: spliceosome network with low cutoff (0.15)**

The leading eigenvalue of the spliceosome network with low cutoff for defining an interaction, which is $\lambda_{A}=60.53$, is much higher than the predictions derived from Erdos-Renyi graphs (analytical prediction: 49.76; numerical simulations: 49.78 $\pm$ 0.07, n=1,000 simulations, *P* < 0.001). As a consequence, even assuming a lower cutoff, the spliceosome network favors a higher path proliferation. For instance, the number of paths with length $\zeta=3$ in the spliceosome network assuming low cutoff (0.15) was 9,340,894, whereas the number of paths expected for random networks were consistently smaller using both null models (Erdos-Renyi graphs = 6,289,465.87$\pm$17,404.75, *P* < 0.001).

In the spliceosome network assuming higher cutoff we detected a concentration of paths starting and finishing within a module. This pattern holds for the description assuming the low cutoff. In fact, although the level of modularity is smaller in the network with low cutoff (Table S1), we still detect a concentration of interactions within modules, as indicated by the clustering coefficient that is larger than network connectance (Table S1) and by within-module concentration of interactions $\delta^{(1)}=1.86$. Again, the values of $\delta^{(\zeta)}$converge to values close to one as $\zeta$ increases, but a concentration of paths within modules similar to observed in the network using high cutoff still observed, $\delta^{(\zeta\to\infty)}=1.02$. As a consequence of the modular structure, the algebraic connectivity of the empirical network assuming a low cutoff (0.986) was much smaller than predicted by the Erdos-Renyi graphs (34.60$\pm$1.51, P < 0.001). Finally, we simulated the protein-failure dynamics using the spliceosome network assuming low cutoff. Again, our mean-field, continuous approximation predicted the transient dynamics of spliceosome collapse (Figure S1). The rate of decay in the numerical simulations of our protein failure model ($t^{-2.11}$) was very similar to the mean-field prediction ($t^{-2.20}$) and close to the predictions based on algebraic connectivity: $t^{e^{\lambda_{L}}}=t^{-2.68}$). In contrast, the exponential rate of decay expected by Erdos-Renyi graphs was $e^{-0.54t}$.

Fig S1. The time-to-collapse of the spliceosome network in a model of failure spreading. Each closed circle is the median number of functional proteins (1000 simulations) per time. Each open circle is the analytical prediction of the number of functional proteins per time derived from a mean-field approximation of the model (1,000 simulations). Grey squares represent the median number of functional proteins using theoretical networks generated by a more conservative null model (1,000 simulations, see main text for further details).

1. **Sensitivity analyses: alternative null model**

We verified all results using an alternative null model in which we preserve the heterogeneity in the number of interactions per protein. In this null model the probability of two proteins to interact is proportional to $\frac{1}{2}\left( \frac{k_{i}}{N}+\frac{k_{j}}{N} \right)$, in which $k_{i}$ ($k_{j}$) is the number of proteins interacting with protein *i* (*j*) and *N* is the number of proteins in the network. Therefore, we are able to control the confounding effects of the distribution of interactions per protein on our analyses on the role of network structure and, especially modularity, in shaping path integration and cascading effects. In short, almost all results reported using Erdos-Renyi graphs hold after using this more selective model.

For example, the leading eigenvalue of the spliceosome network was lower than the leading eigenvalue of the networks generated by this second null model (high cutoff = $25.84$, null model = 19.86$\pm0.57$, *P* < 0.001; low cutoff = $60.53$, null model = 51.93$\pm0.68$, *P* < 0.001). As a consequence, path proliferation was higher in the empirical networks than in the null model networks (number of paths with length $\zeta=3$; high cutoff = $602,250$, null model = 346,556.40$\pm$29,282.10; low cutoff = 9,340,894, null model = 6,815,610.36$\pm265,771.03$, *P* < 0.001). Accordingly, the algebraic connectivity of the spliceosome network was smaller than predicted by this alternative null model (high cutoff = $0.689$, null model = 4.16$\pm$1.02, *P* < 0.001; low cutoff = 0.986, null model = 19.55$\pm$2.20, *P* < 0.001). Finally, we simulated the dynamics of protein failure using the theoretical networks generated by this second null model. The number of functional proteins decays exponentially (high cutoff null model =$e^{-0.54t}$; low cutoff null model = $e^{-1.09t}$), contrasting with the power-law decay observed in the simulations using the empirical network (high cutoff = $t^{-1.80}$; low cutoff = $t^{-2.11}$).
